# Association between low birth weight and impaired glucose tolerance in children: a systematic review and meta-analysis

**DOI:** 10.3389/fped.2024.1362076

**Published:** 2024-05-09

**Authors:** Jun Ma, Youfang Wang, Mengyan Mo, Zerong Lian

**Affiliations:** ^1^ Department of Pediatrics, Heping Hospital Affiliated to Changzhi Medical College, Changzhi, Shanxi, China; ^2^ Department of Gastroenterology, Guangzhou Red Cross Hospital, Guangzhou, Guangdong, China; ^3^ Department of Otolaryngology, Heping Hospital Affiliated to Changzhi Medical College, Changzhi, Shanxi, China; ^4^ Department of Nursing, Heping Hospital Affiliated to Changzhi Medical College, Changzhi, Shanxi, China

**Keywords:** newborn, low birth weight, abnormal glucose tolerance, diabetes, meta-analysis

## Abstract

**Background:**

A potential association between the onset of diabetes and normal birth weight (NBW) has been discovered. Diverse conclusions and study methodologies exist regarding the connection between low birth weight (LBW) and impaired glucose tolerance in children, underscoring the need for further robust research. Our institution is embarking on this study to thoroughly examine the association between LBW and impaired glucose tolerance in children.

**Methods:**

We conducted searches on Cochrane Library, ScienceDirect, EMBASE, PubMed, China National Knowledge Infrastructure (CNKI), Chinese Biomedical Literature data (CBM) online database, VIP full-text Database, and Wanfang Database to identify correlation analyses or case-control studies investigating the relationship between LBW and abnormal glucose tolerance in children. The search spanned from January 2010 to September 2023. The quality of observational studies was evaluated using the Newcastle–Ottawa Scale (NOS) tool. Data synthesis was performed using the statistical software RevMan 5.3 for meta-analysis.

**Results:**

Based on the preferred reporting items for systematic reviews and meta-analysis (PRISMA) guidelines, we finally included 10 clinical control studies consisting of a total of 2971 cases. There wasn’t considerably change in blood sugar levels among LBW, NBW and high birth weight (HBW) infants (*P *> 0.05). There was no significant difference in insulin levels between LBW infants and NBW infants (*P *> 0.05). The HOMA-IR of LBW infants was considerably higher than that of NBW infants (*P *< 0.05). The risk of abnormal glucose tolerance in LBW infants was 0.42 times higher than that in NBW and HBW infants [Fisher's *Z* = 0.42, 95% CI = (0.09, 0.75), *P *= 0.01].

**Conclusion:**

LBW is associated with an increased risk of abnormal glucose tolerance, as indicated by elevated HOMA-IR level in LBW infants compared to NBW and HBW pediatric population. Further research is needed to confirm and expand upon these findings to better understand the complex relationship between LBW and impaired glucose tolerance in children.

## 1 Introduction

In China, the prevalence of diabetes has surged, with over 30 million individuals affected, marking a substantial rise from 0.8% in 1980 to 3.5% in 2000 (1, 2). A study conducted from 2015 to 2017 revealed that in China, the overall prevalence of diabetes among adults is 12.8%, including a newly diagnosed diabetes prevalence of 6.8% and a self-reported diabetes prevalence of 6.0% (3–6). The rising incidence of diabetes has led to an increased prevalence of the condition among young adults, and reports indicate that diabetes can manifest in individuals as young as 13 years old (7, 8). The presence of concurrent complications such as hyperlipidemia, hypertension, and other conditions alongside diabetes has garnered increasing attention in terms of the onset, progression, outcomes, and management of diabetes. Diabetic complications are more common in macrovascular and microvascular diseases, and abnormal blood lipid metabolism is involved in the whole process of this disease. A randomized controlled trial study has demonstrated the intricate interplay between blood glucose and blood lipids in individuals with diabetes (9–11).

Given the rising incidence and prevalence of type 2 diabetes among children and adolescents, this issue may emerge as a significant public health concern impacting both developed and developing nations. Consequently, from a population standpoint, it is imperative to identify potential risk factors and identify susceptible groups that could benefit from screening and preventive measures (12–14). So far, scholars have explored the etiology of diabetes from various perspectives and directions, including pathology, genetics, genomics, social factors, and other fields. The formation of diabetes corresponds to abnormal birth weight (15, 16). The occurrence of high birth weight infants, often stemming from fetal overnutrition, maternal diabetes, and other maternal health conditions, can significantly predispose individuals to obesity and diabetes in adulthood, typically around the age of 18. This association may be attributed to genetic polymorphisms and the onset of insulin resistance (17). Additionally, abnormal insulin secretion during the fetal period, impacting fetal growth and development, may contribute to the prevalence of infants with low birth weight (LBW) and heighten the risk of diabetes in adulthood (18, 19).

At present, numerous investigations have explored the link between LBW and impaired glucose tolerance in children. However, these studies yield varying conclusions and employ designs, leading to poor applicability. The findings of a single study regarding the correlation between LBW and impaired glucose tolerance in children may lack conviction without robust scientific support. Therefore, additional research is warranted, necessitating reputable scientific studies to comprehensively evaluate this relationship. Consequently, a thorough, quantitative, and systematic meta-analysis of independent studies with similar objectives was conducted to investigate the association between LBW and impaired glucose tolerance in children. This analysis aims to provide valuable insights to inform further exploration of the underlying causes of type 2 diabetes and to enhance eugenic strategies.

## 2 Methods

### 2.1 Database and literature search

A computer-based search was carried out across multiple databases, including CochraneLibrary, ScienceDirect, EMBASE, Wanfang Database, the Chinese Biomedical Literature Data (CBM), VIP Full-text Database, China National Knowledge Infrastructure (CNKI). This extensive search strategy encompassed a wide range of sources, including both degree papers, conference papers, Chinese and foreign periodicals, news articles, and manual searches, among others.

The main aim was to collect pertinent data regarding the association between LBW and impaired glucose tolerance in children. The literature retrieval process utilized a combination of free-text and subject-specific keywords. Key search terms such as “newborn,” “low birth weight,” and “impaired glucose tolerance” were employed, with the search period spanning from January 2010 onwards. This comprehensive strategy aimed to encompass the latest and most relevant research findings in the field.

### 2.2 Inclusion criteria and exclusion criteria

#### 2.2.1 Criteria for include literature

(1) Observational studies that were published in full-text format.
(2) Inclusion of newborns with birth weight of less than 1,500 g.
(3) Assessment of the correlation between LBW and impaired glucose tolerance in children.
(4) Adjustment or control for the potential confounding factors, with the reporting of relative risk factors or the comparison of blood glucose, insulin, and Model Assessment for Insulin Resistance (HOMA-IR) indices with those of normal newborns and high-birth-weight newborns. Based on a previous literature (20), children were classified into LBW (<2,500 g), normal birth weight (NBW; 2,500–3,999 g), and high birth weight (HBW; ≥4,000 g). Impaired glucose tolerance was defined as having 2-h plasma glucose concentration (2hPG) 140–199 mg/dl (21).

#### 2.2.2 The literature exclusion criteria

(1) Studies with incomplete and unusable data.
(2) Duplicate research content, with preference given to the most recent study.
(3) Reviews, editorials, preclinical studies, and literature that did not directly relate to the special purpose of the current meta-analysis.
(4) Clinical cases, which were not considered in this particular meta-analysis.

### 2.3 Study selection and data extraction

The process of extracting data and screening books followed a rigorous and systematic approach.

#### 2.3.1 Independent screening

Two researchers conducted separate reviews of the selected literature and extracted relevant information.

#### 2.3.2 Quality evaluation

These researchers also assessed the quality of the included studies.

#### 2.3.3 Cross-check

To ensure accuracy and consistency, the results of the independent screenings and data extractions were cross-checked. Any discrepancies were addressed through discussion and consensus. In instances of unresolved discrepancies, a third researcher was consulted to provide adjudication.

#### 2.3.4 Software utilization

NoteExpress document management software and Excel office software were employed for data management and extraction, facilitating efficient organization and analysis of the research data.

#### 2.3.5 Data completeness

In cases where the literature lacked necessary information, the authors of the respective articles were contacted to request Supplementary Data.

The information retrieved from the data comprised: (1) the authors’ names, the publishing year and the country of the institute; (2) the characteristics of the study design; (3) the characteristics of participants, including health status, sample size and average age; (4) the number of normal weight, overweight and LBW newborns; and (5) confounding factors adjusted or controlled when reporting correlations.

### 2.4 Qualitative assessment

For assessing the quality of observational studies in this meta-analysis, the Newcastle-Ottawa Scale (NOS) tool was utilized. Studies with a NOS score of ≥6 were categorized as medium to high quality, whereas those with an NOS score <6 were classified as low quality.

### 2.5 Statistical analysis

RevMan 5.3 software, derived from the Cochrane Collaboration, was used for conducting meta-analyses. The mean values, and standard deviations for Blood glucose levels, insulin levels, HOMA-IR in each group were input into RevMan 5.3 for analysis. The weighted mean difference (WMD) was used as the effect size, and 95% confidence intervals (CI) were calculated. Heterogeneity was evaluated using the *χ*^2^ test and the *I*^2^ statistic, which quantifies the total variation across studies attributed to heterogeneity. *P*-value below 0.05 was deemed statistically significant (22, 23).

## 3 Results and analysis

### 3.1 The outcomes of literature retrieval and the fundamental circumstances behind literature inclusion

In adherence to the Preferred Reporting Items for Systematic Review and Meta-analysis (PRISMA) guidelines, the study initiated with a computer-based database search, resulting in the retrieval of 742 studies. After eliminating duplicate studies, 561 unique studies remained. These papers were then subject to preliminary screening, during which 308 studies were reviewed.

**Figure 1:**
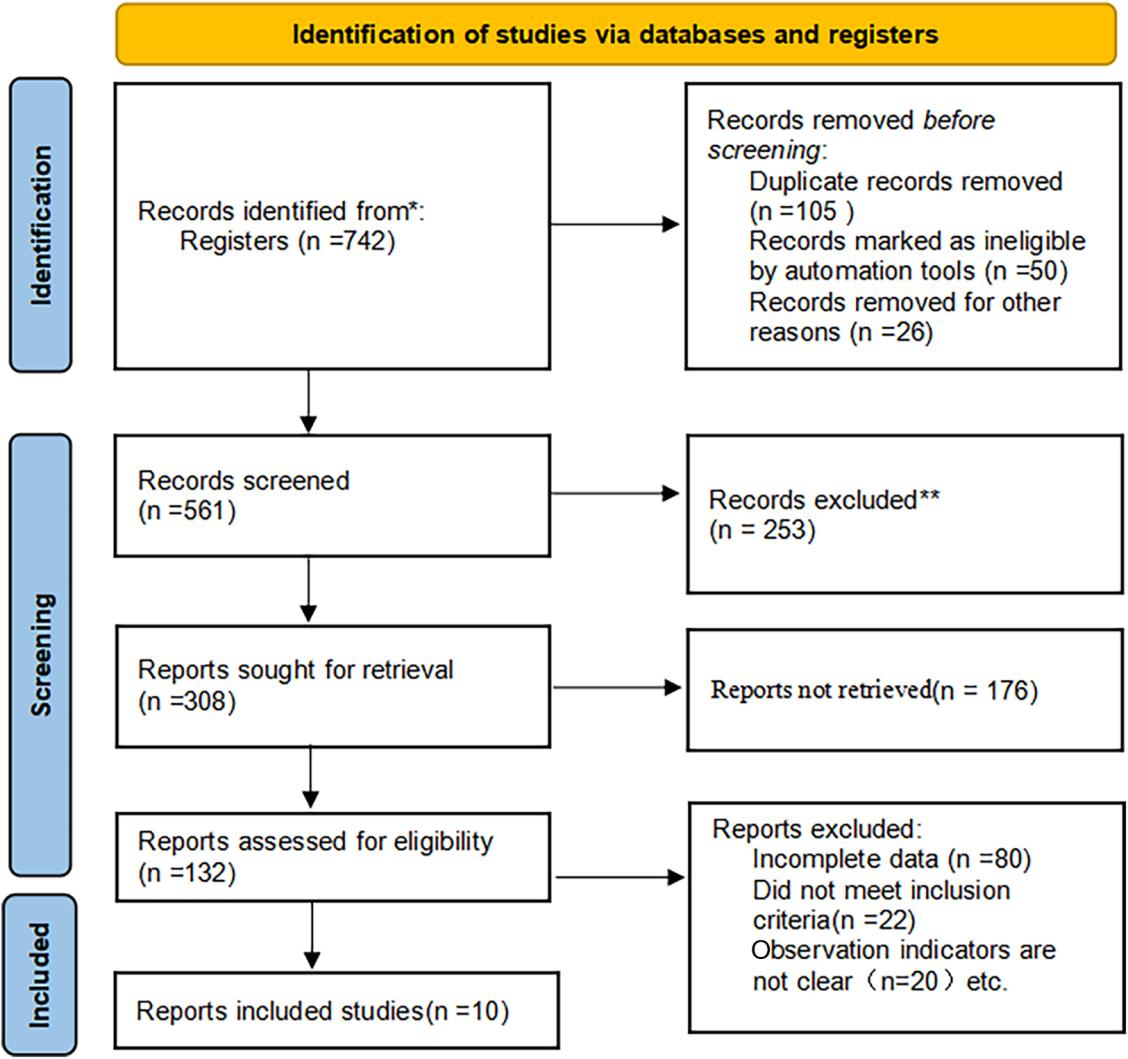
Illustration of literature screening.

After the initial screening, 142 studies met the inclusion criteria for further assessment, while irrelevant studies, reviews, case reports, and uncontrolled documents were excluded. Subsequently, the full texts of the selected literature underwent thorough examination, with papers containing incomplete data or lacking key outcome indicators being excluded. Ultimately, the study integrated data from 10 clinical control studies, comprising a total of 2,971 samples. This meticulous selection process ensured that the included studies were pertinent, met the required criteria, and enhanced the robustness of the meta-analysis. Figure 1 illustrates the flow chart detailing the literature screening process, while Table 1 presents the fundamental characteristics identified in the literature.

**Table 1:** Basic characteristics of literature.

Include the literature	Year of publication	Country	Sample size			Age (years)	Outcome index
			LBW	NBW	HBW		
Oliveira-Santos et al. (24)	2019	Portugal	27	366	22	14.1 ± 1.7	③④
Ledo DL et al. (20)	2018	Brazil	40	600	79	9.5 ± 2.0	①②③④
De Jong M et al. (25)	2023	Holland	32	82	/	Not available	②③④
Dominguez-Hemandez C et al. (26)	2015	Mexico	33	41	/	10.1 ± 1.8	①②③④
dos Santos Alves PJ et al. (27)	2015	America	86	86	/	13.2 ± 2.5	①
Mori M et al. (28)	2012	Japan	13	34	/	15.4 ± 1.4	①②③④
Guerrero-Romero F et al. (29)	2010	Mexico	77	1088	97	11.8 ± 2.2	①
Huang Y et al. (30)	2015	China	30	30	/	6.5 ± 2.5	④
Sebastiani G et al. (31)	2015	Spain	19	27	/	Not available	④
Bluskova Z et al. (32)	2014	Russia	31	31	/	Not available	④

①Blood glucose level; ②Insulin level; ③HOMA-IR; ④Relativity analysis.

### 3.2 An assessment of the study’s methodology’s quality

All the literatures described the detailed intervention methods and observation indicators, and all the literatures did not describe the quantity and causes of blind procedures, as well as missed follow-up or withdrawal, in detail. The NOS scale study indicated that low-quality literature had a score of <6, while high-quality literature had a score of ≥6 (Table 2).

**Table 2:** Literature quality.

Study	Representativeness of the exposed cohort	Selection of the nonexposed cohort	Selection		Comparability		Outcome		Quality score
			Ascertainment of exposure	Demonstration that outcome of interest was not present at start of study	Comparability of cohorts on the basis of the design or analysis	Assessment of outcome	Was follow-up long enough for outcomes to occur	Adequacy of follow up of cohorts	
Oliveira-Santos et al. (24)	★	★	★	★	☆	★	★★	★	8
Ledo DL et al. (20)	★	★	★	★	★	★	☆	☆	6
De Jong M et al. (25)	☆	★	★	★	★	☆	★	☆	6
Dominguez-Hemandez C et al. (26)	☆	★	★	★	★	★	☆	★	6
dos Santos Alves PJ et al. (27)	★	★	★	★	★★	★	☆	★	9
Mori M et al. (28)	★	★	★	★	★	★	★	☆	7
Guerrero-Romero F et al. (29)	★	☆	★	★	★	☆	★	★	6
Huang Y et al. (30)	☆	☆	★	★	★	★	☆	★	6
Sebastiani G et al. (31)	☆	☆	★	★	★	★	★	★	7
Bluskova Z et al. (32)	★	★	★	★	★	★	☆	★	7

“★” = 1 point of the Newcastle-Ottawa scale, studies get 1point at each category once they met the criteria; “☆” = 0 point of the Newcastle-Ottawa scale.

### 3.3 Meta analysis result

#### 3.3.1 Blood glucose level

**Figure 2:**
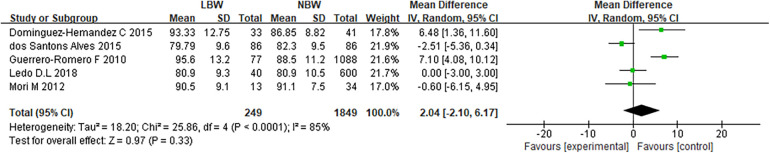
Comparison of blood glucose levels between normal weight and low birth weight children forest analysis chart F.

**Figure 3:**
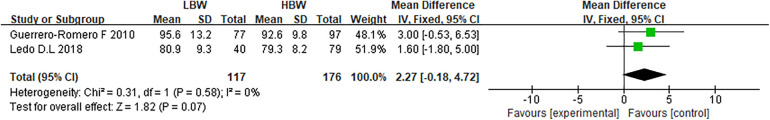
Comparison of blood glucose levels between overweight and low birth weight children forest analysis map.

The blood glucose levels of each group were examined using meta, and the heterogeneity test results revealed that LBW vs. NBW: Chi^2^ = 25.86, *I*^2^ = 85%, *P *< 0.0001, df = 4; LBW vs. HBW: Chi^2 ^= 0.31, *I*^2 ^= 0%, *P *= 0.58, df = 1. From the analysis shown as Figures 2, 3, there was no statistical difference in blood sugar levels between LBW infants and normal weight and overweight infants (*P* > 0.05).

#### 3.3.2 Insulin level

**Figure 4:**
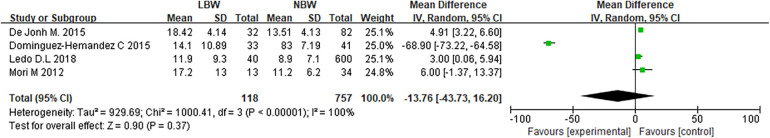
Comparison of insulin levels between normal weight and low birth weight children forest analysis map.

A meta-analysis of the comparative results of insulin levels was performed in each group. In the comparison between LBW and NBW, with four degrees of freedom, the Chi^2^ statistic yielded a value of 6.85, resulting in a *p*-value of 0.14 and an *I*^2^ of 42%. These findings indicate moderate heterogeneity among the studies for this comparison. In the comparison between LBW and HBW, the Chi-squared value was 11.78 with one degree of freedom, resulting in a *p*-value of 0.0006, and *I*^2^ was determined to be 92%. These results indicate a high level of heterogeneity among the studies for this comparison. According to the analysis of the random-effect model (Figure 4), there wasn't considerably difference in insulin level between LBW infants and normal weight children (*P *> 0.05).

#### 3.3.3 HOMA-IR

**Figure 5:**
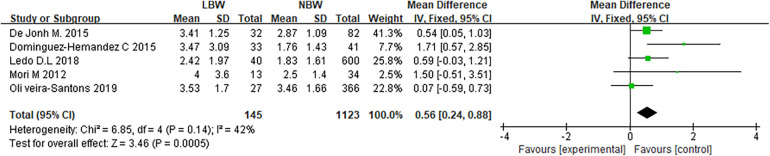
Forest analysis map of HOMA-IR comparison between normal weight and low birth weight children.

In the comparison between LBW and normal birth weight (NBW) children (Figure 5), with four degrees of freedom, the Chi-squared statistic was 6.85, yielding a *p*-value of 0.14 and an *I*^2^ of 42%, indicating a moderate level of heterogeneity among the studies.

**Figure 6:**
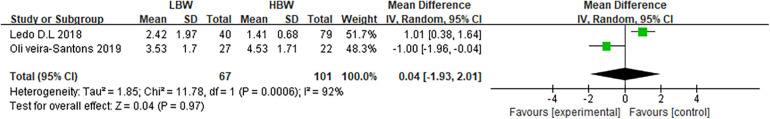
Forest analysis map of HOMA-IR comparison between overweight and low birth weight children.

In the comparison of LBW with HBW children (Figure 6), with one degree of freedom, the Chi-squared statistic was 11.78, yielding a *p*-value of 0.0006, and *I*^2^ was determined to be 92%, suggesting a high level of heterogeneity among the studies for this comparison. The meta-analysis findings reveal that LBW infants have significantly higher HOMA-IR values when compared to NBW children (*P *< 0.05). Nonetheless, in comparing LBW to HBW children, the observed high level of heterogeneity underscores the need for caution in interpreting the results. This heterogeneity indicates significant variability among the included studies in this comparison, potentially influencing the overall findings.

#### 3.3.4 Analysis of correlation between low birth weight and HOMA-IR

**Figure 7:**
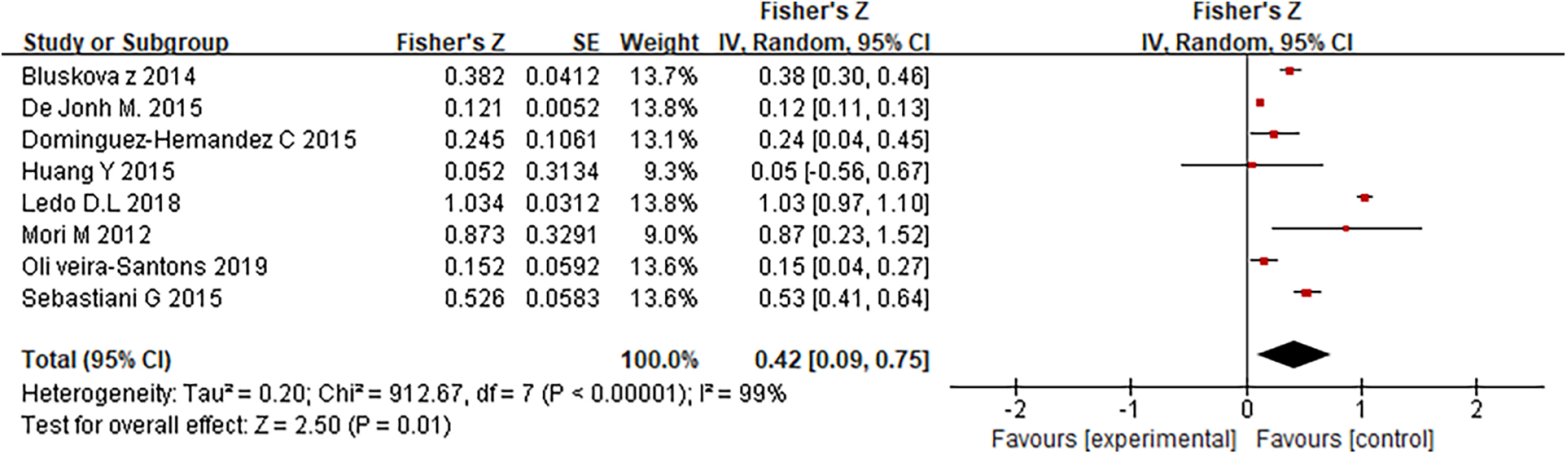
Forest analysis map of the correlation between low birth weight and HOMA-IR.

This study encompassed data from 10 clinical controlled studies, comprising a total of 2,971 samples, and conducted a meta-analysis on the association between LBW and HOMA-IR. The heterogeneity test results indicated significant heterogeneity, with Chi^2 ^= 912.67, df = 7, *P* < 0.00001, and *I*^2 ^= 99%. These findings suggest a substantial level of variation among the included studies’ meta-analyses, assessed using a random effects model (Figure 7), the risk of abnormal glucose tolerance in LBW newborns was 0.42 times higher than that in normal and overweight children [Fisher's *Z* = 0.42,95% CI:0.09–0.75, *P *= 0.01].

#### 3.3.5 Publication bias analysis

The funnel diagram was created using the blood glucose, insulin level, HOMA-IR value and correlation analysis results of each group, and an examination of publication bias was conducted (Supplementary Figures S1–S4). The results revealed that while a small proportion of the included studies exhibited asymmetry, the majority of funnel plots appeared symmetrical, suggesting potential publication bias in the included literature. This bias could be linked to the heterogeneity observed in the study.

## 4 Analysis and discussion

Previous research has shown a link between diabetes and LBW (33). The “Fetal Origin hypothesis,” proposed in the 1990s, suggests that the conditions experienced during fetal intrauterine development significantly influence the risk of developing diseases in adulthood. According to this hypothesis, individuals born with LBW are at a considerably higher risk of developing type 2 diabetes later in life (34). Preterm delivery or intrauterine growth restriction is the most common cause of LBW (35). 63% of LBW infants are born prematurely, while the remaining cases are attributed to intrauterine dysplasia. It is noteworthy that nearly all very low birth weight infants are born prematurely, with some being extremely premature, with gestational ages of less than 25 weeks. In utero stunting of development in LBW infants impairs the development and function of the pancreas, leading to problems with lipid and glucose metabolism and hypertension in adulthood (36, 37). Genetic research indicates that variations in susceptibility genes associated with type 2 diabetes may also be linked to LBW. This suggests a potential genetic predisposition for both lower birth weight and an increased risk of type 2 diabetes later in life. These findings underscore the intricate interplay between genetic factors and health outcomes across the lifespan (38). If an individual has a low birth weight or childhood weight, there is a tendency for rapid weight gain in adulthood (after 18 years of age) due to dietary changes, which significantly increases the risk of developing diabetes and other related metabolic disorders. Reduced birth weight has been associated with the upregulation of certain genes, commonly known as “thrift genes.” These genes might be involved in metabolic adaptations to prenatal undernutrition. Furthermore, there is evidence connecting LBW to a higher risk of developing several disorders, including diabetes, in adulthood, suggesting that early life factors, including birth weight, can influence gene expression and can aid in the later-life development of chronic illnesses.

Recently, LBW infants are prone to developing obesity, insulin resistance, hypertension, and vascular diseases in adulthood. Additionally, the incidence and mortality rates of other conditions such as enterocolitis, late-onset septicemia, and intraventricular hemorrhage are elevated in this population (39). The prevalence of diabetes and hypertension in LBW infants heightened significantly in adulthood. A survey has shown that the incidence of type 2 diabetes and birth weight are correlated in a U-shaped manner, and the quantity of diabetes cases complicated with hypertension in LBW is significantly increased. Diabetes is also associated with high birth weight, while hypertension is notably more prevalent among high birth weight infants. It is hypothesized that hypertension in high birth weight infants and LBW infants may arise from distinct metabolic phenotypes or similar environmental factors. Moreover, LBW infants exhibit a significantly higher prevalence of hyperlipidemia compared to those with normal birth weight (40). Previous study has found that 300 cases of high birth weight infants, and the results show the detection rates of overweight and obesity in the macrosomia group (13.10% vs. 2.86%) are higher than those in the control group (9.69% vs. 1.61%) (41), which suggested that the risk of insulin resistance and abnormal lipid metabolism in abnormal birth weight infants is greater than that in normal birth weight infants. China's Chinese multi-provincial Study on Risk Factors of Cardiovascular Diseases (CMCS) has suggested that the proportion of diabetic patients with abnormal blood lipid metabolism is considerably higher, and the proportion of diabetic patients with atherosclerosis risk factors such as coronary heart disease, cerebral infarction and venous thrombosis is also significantly higher than that of non-diabetic patients.

More and more evidence shows that the LBW of newborns is directly related to the abnormal glucose tolerance of children. The blood sugar and insulin levels of LBW newborns, normal newborns and overweight newborns were analyzed by meta-analysis. The findings indicated that there was not a significant variation between the blood sugar levels of LBW newborns and overweight and normal newborns. Meta-analysis of the comparison results of HOMA-RI values in each group showed that the HOMA-IR values of LBW infants were considerably higher. It is suggested that there is a certain correlation between LBW of newborns and HOMA-IR. Meta-analysis was made on the correlation between LBW and HOMA-IR, and random effect model analysis showed the risk of abnormal glucose tolerance in LBW newborns was 0.42 times higher than that in normal and overweight children [Fisher's SZ = 0.42, *P* = 0.01, 95%CI = (0.09, 0.75)]. Through an analysis of existing research in this domain, it is evident that there exists a connection between abnormal glucose tolerance and atypical birth weight in LBW infants. This association cannot be solely attributed to factors related to the fetus itself, prenatal malnutrition, or the intrauterine environment; rather, it encompasses various other contributing factors. These factors encompass aspects related to the pregnant woman's health, as well as lifestyle choices and dietary habits during adulthood. Additionally, genetic modifications resulting from certain factors in adulthood may also influence this intricate relationship. Understanding these multifaceted connections is crucial for comprehensively addressing and managing health risks associated with abnormal glucose tolerance and birth weight.

However, the study has certain limitations that warrant consideration:

(1) Stringent Criteria for Inclusion and Exclusion: The study employed rigorous criteria for inclusion and exclusion, leading to a relatively small number of included studies. Furthermore, detailed subgroup analysis was not conducted on studies displaying heterogeneity. This limited the diversity of the included literature and may affect the generalizability of the findings.
(2) Inconsistent Treatment Protocols and Outcome Measures: Variability in the treatment protocols and outcome indicators across the included studies may introduce heterogeneity and impact the reliability of the outcomes.For example, insulin level is influenced by age and gender (42). Therefore, these factors may influence the results in this study. To bolster the robustness of the findings, it is imperative to conduct further research, encompassing high-quality correlation studies and case-control trials. These endeavors will provide a deeper understanding of the relationship between abnormal glucose tolerance and birth weight, thus advancing our knowledge in this critical area of study.

## 5 Conclusion

It has been shown that LBW in babies is associated with poor glucose tolerance in pediatric population and a higher chance of type 2 diabetes in adults. This underscores the significance of preventive measures to manage birth weight abnormalities.Highlighting the significance of dietary and exercise management during the perinatal and developmental stages is crucial for mitigating the risk of diabetes. These insights underscore the necessity of early interventions and a comprehensive healthcare approach to mitigate the enduring adverse impacts of low birth weight on health outcomes.

## Data Availability

The datasets used and analyzed during the current study available from the corresponding author on reasonable request.

## Supplementary material

Supplementary Figure S1 Funnel chart based on blood glucose level. Note: (**A**) Comparison between LBW and DBW; (**B**) LBW compared with HBW.

Supplementary Figure S2 Funnel chart based on insulin level.

Supplementary Figure S3 Funnel diagram based on HOMA-IR. Note: (**C**) LBW compared with DBW; (**D**) LBW compared with HBW.

Supplementary Figure S4 Funnel chart based on the results of correlation analysis.
