# Supplementary figures and images for: Association between low birth weight and impaired glucose tolerance in children: a systematic review and meta-analysis
Source: Front Pediatr. 2024 May 9;12:1362076. doi: 10.3389/fped.2024.1362076 (PMC11112083; doi:10.3389/fped.2024.1362076)

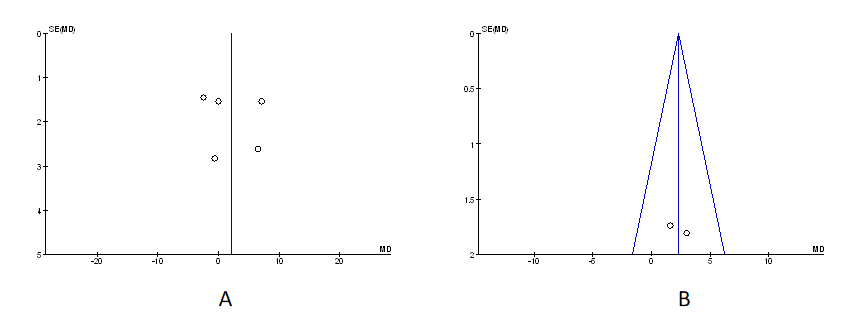

Supplement: Supplementary Figure S1 — Funnel chart based on blood glucose level. Note: (A) Comparison between LBW and DBW; (B) LBW compared with HBW. [file Image1.tif]

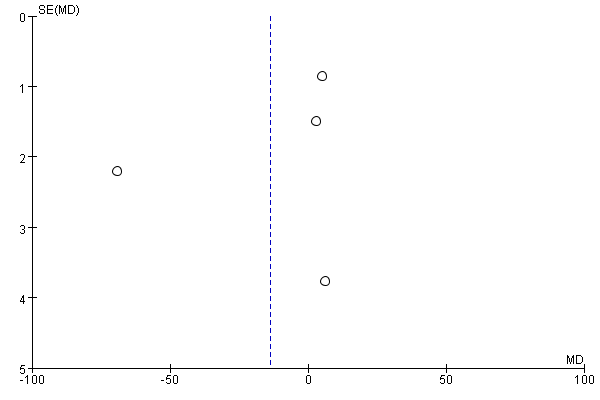

Supplement: Supplementary Figure S2 — Funnel chart based on insulin level. [file Image2.tif]

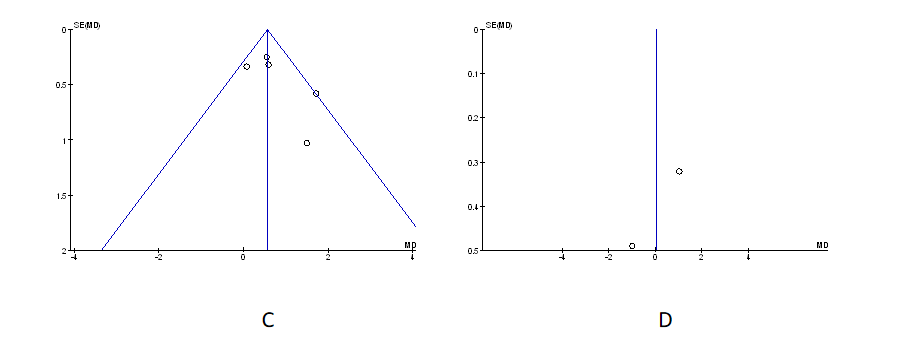

Supplement: Supplementary Figure S3 — Funnel diagram based on HOMA-IR. Note: (C) LBW compared with DBW; (D) LBW compared with HBW. [file Image3.tif]

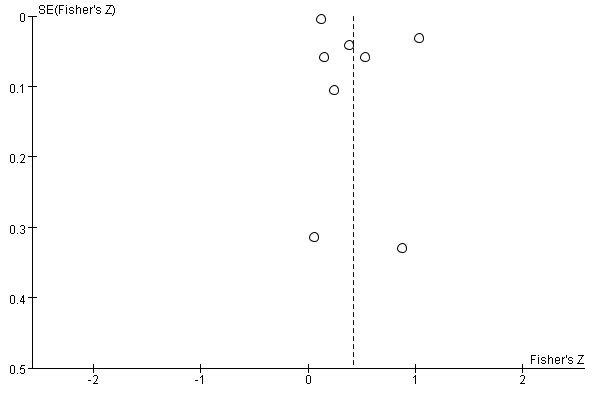

Supplement: Supplementary Figure S4 — Funnel chart based on the results of correlation analysis. [file Image4.tif]
